# Lipoprotein Profiles before Heparin Administration in Patients with or without Coronary Thrombosis Following Atherosclerosis

**DOI:** 10.3400/avd.oa.20-00146

**Published:** 2021-03-25

**Authors:** Tomomi Koizumi, Hideaki Kaneda, Nobuyuki Komiyama, Ikuo Inoue, Toshihiro Muramatsu, Katsuyuki Nakajima

**Affiliations:** 1 Department of Cardiovascular Medicine, Mito Medical Center, National Hospital Organization, Higashi-Ibaraki, Ibaraki, Japan; 2 Okinaka Memorial Institute for Medical Research, Tokyo, Japan; 3 Cardiovascular Center, St. Luke Hospital, Tokyo, Japan; 4 Department of Endocrinology, Saitama Medical University, Moro, Saitama, Japan; 5 Department of Cardiology, Saitama International Medical Center, Hidaka, Saitama, Japan; 6 Laboratory of Clinical Nutrition and Medicine, Kagawa Nutrition University, Tokyo, Japan

**Keywords:** heparin, lipoprotein, thrombosis, polyacrylamide gel electrophoresis, coronary artery disease

## Abstract

**Objective:** The correlation between lipoproteins and arterial thrombosis is not fully elucidated, and no data exist in terms of lipoprotein profiles before heparin administration in patients with coronary arterial thrombosis (CAT). This cross-sectional study aimed to evaluate the lipoprotein profile before heparin administration in 63 ST-segment elevation myocardial infarction (STEMI) patients with CAT.

**Methods:** The lipoprotein profile was measured via polyacrylamide gel electrophoresis prior to heparin administration for primary percutaneous coronary intervention for STEMI. Age- and sex-matched subjects with <25% stenosis in stable coronary artery disease were enrolled as controls.

**Results:** In the pre-heparin serum, the fraction of very-low-density lipoprotein (P=0.75) in STEMI patients was not different from that in controls, and the fraction of intermediate-density lipoprotein (P<0.01) in STEMI patients was significantly lower than that in controls. Although the fraction of small dense low-density lipoprotein (s-LDL) in STEMI patients was significantly higher than that in controls (P<0.01), 44% (28/63) of STEMI patients were negative for s-LDL.

**Conclusion:** Although lipoproteins are a risk factor for atherosclerosis, lipoprotein profiles with CAT following atherosclerosis in STEMI are different from those profiles without CAT in stable coronary artery disease.

## Introduction

Not all atherosclerosis plaques that progress by lipoproteins promote lethal arterial thrombosis.^1)^ Although the interactions between lipoproteins and platelets have been investigated,^2–7)^ there is no data on lipoprotein profiles at the onset of arterial thrombosis, where platelet aggregation is activated. When evaluating serum lipoprotein profiles in patients with coronary thrombosis leading to acute coronary syndrome (ACS), the effect of heparin on lipoproteins should be considered. As heparin has been reported as a lipid-modifying agent^8–10)^ and is currently considered to be mandatory for the treatment of unstable coronary thrombus in the initial treatment of ACS, heparin administration often starts in the emergency room. Previous published papers on lipoproteins in ACS patients^11–15)^ did not describe the use of heparin before blood collection or the status of coronary arteries in control patients. Heparin has been reported to release hepatic triglyceride lipase (HTGL) from the liver and lipoprotein lipase (LPL) from the endothelial cells into the blood circulation.^16)^ In turn, those lipases play a significant role in lipid metabolism by hydrolyzing triglycerides (TG) and phospholipids in circulating lipoproteins.^17)^ The present study aimed to determine pre-heparin administration lipoprotein profiles at the onset of coronary arterial thrombosis (CAT) in patients with ST-segment elevation myocardial infarction (STEMI). It also aimed to compare these profiles with those of stable patients with mild coronary atherosclerosis without CAT via polyacrylamide gel electrophoresis (PAGE).

## Materials and Methods

### Study population

The present cross-sectional study recruited 108 consecutive STEMI patients within 12 h from symptom onset, who had been admitted to the Saitama Medical University International Medical Center from February 2011 to November 2012. STEMI was characterized by persistent chest pain at rest, accompanied by new or presumed-new ST-segment elevation in>2 contiguous leads with a cutoff point of ≥0.2 mV. Patients who had angiographic documentation of stenosis of >90% of at least one major coronary artery via CAT were included. CAT was defined as thrombus documented in infarct-related artery with thrombolysis in myocardial infarction (TIMI) Coronary Grade Flow 0 or 1 by coronary cineangiography: Grade 0, no antegrade flow beyond the point of occlusion; Grade 1, the contrast material passes beyond the area of obstruction but “hangs up” and fails to opacify the entire coronary bed distal to the obstruction for the duration of the cineangiographic filming sequence.^18)^ A total of 45 patients were excluded as they met one of the following exclusion criteria: history of prior statin or other lipid-modifying therapy, no chance to obtain blood sample before heparin administration, unstable angina pectoris or non-STEMI, age >85 years, autoimmune disease, pancreatitis, hepatic or renal dysfunction, active malignancy, infectious disease, cardiogenic shock, overt congestive heart failure, prior myocardial infarction, stroke, previous coronary artery bypass graft surgery, and previous percutaneous coronary intervention (PCI). Of the 108 patients recruited, 63 were finally included in this study. This study also enrolled consecutive control subjects who underwent elective cardiac catheterization for atypical chest pain with electrocardiogram changes, valvular heart disease before surgical treatment, or cardiomyopathy during the same study period as the patients with suspected coronary artery disease. The control subjects had angiographically mild coronary atherosclerosis (<25% stenosis) and no clinical evidence of coronary artery spasm. Of these subjects, this study finally included age-matched (±3 year) and sex-matched (n=63) control subjects: suspected coronary artery disease but coronary artery <25% stenosis (n=50), valvular heart disease (n=8), or cardiomyopathy (n=5), who did not have any of the same exclusion criteria as described above.

Patients were pretreated orally with 200 mg of aspirin and intravenous heparin, which were administered immediately before revascularization. Anticoagulation was maintained as an activated clotting time of 250 to 300 s during PCI.

The medications after primary PCI were as follows: aspirin 100 mg daily, clopidogrel 75 mg daily, beta-blockers, angiotensin-converting enzyme inhibitor, or angiotensin II receptor blocker. Statins were administered following blood sample collection post-PCI.

### Collection of blood

**Figure 1:**
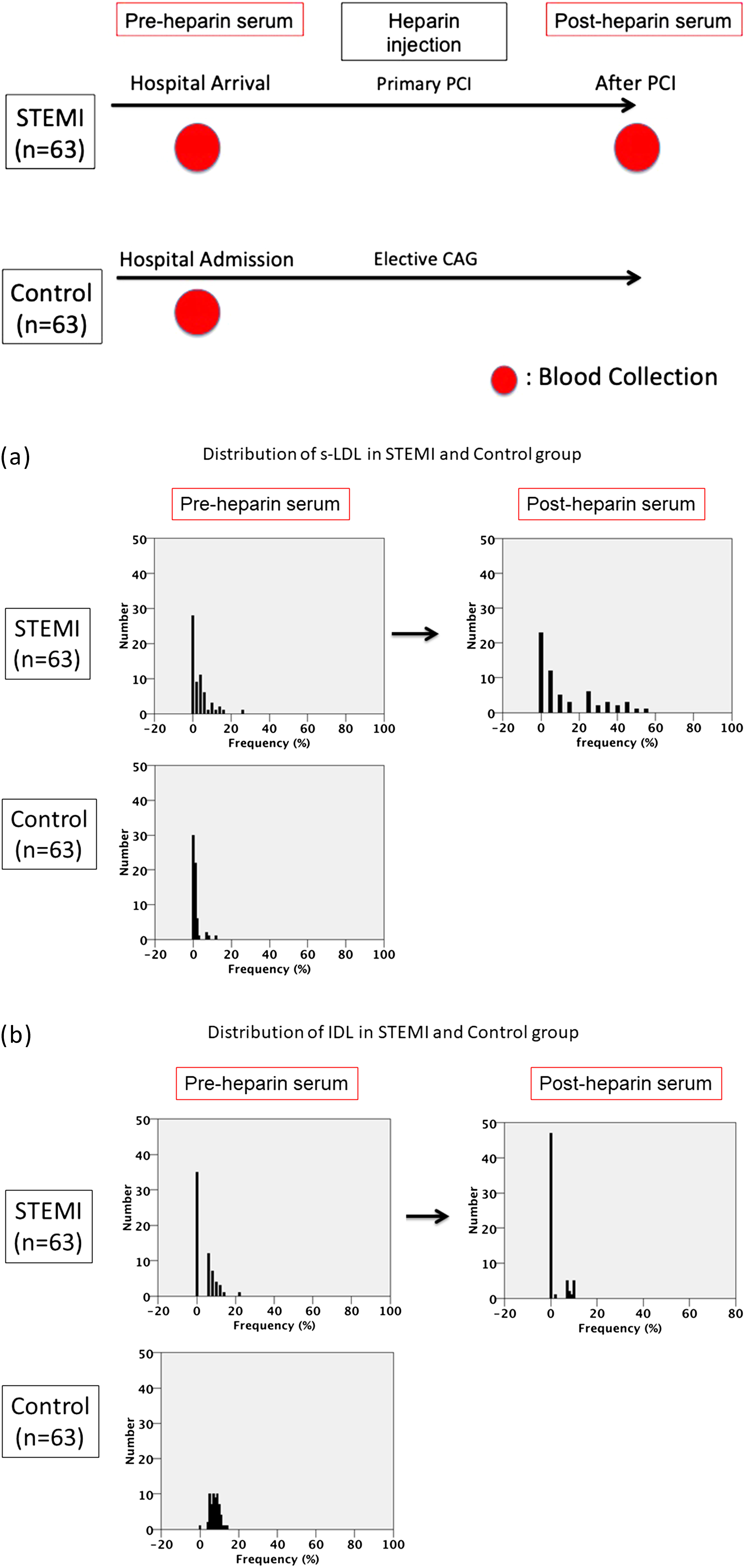
Fig. 1 Time points of blood collection. The red circle indicates the time point of blood collection. Blood samples were collected upon arrival at the hospital before any drug, including heparin, was administered to STEMI patients. After PCI, blood samples were collected from the artery before statin administration to STEMI patients, where the activated clotting time was controlled from 250 to 300 s. For the control patients, blood samples were collected before heparin administration in elective diagnostic coronary cineangiography. Blood samples were not collected after coronary cineangiography in the control patients. (**a**) Cumulative relative frequency plots of s-LDL in the STEMI and control groups are presented in **Fig. 1a**. Note that about half of the STEMI group in pre-heparin serum is negative for s-LDL. (**b**) Cumulative relative frequency plots of IDL in the STEMI and control groups are presented in **Fig. 1b**. Note that 55% (35/64) of the STEMI group in the pre-heparin serum is negative for IDL.

CAG: cineangiography; IDL: intermediate-density lipoprotein; PCI: primary percutaneous coronary intervention; s-LDL: small, dense low-density lipoprotein; STEMI: ST-segment elevation myocardial infarction

Blood samples were collected from the patients’ femoral artery upon arrival at the hospital and before administration of any drug. Immediately after PCI, blood samples were collected, and the activated clotting time was still controlled from 250 to 300 s. For the control patients, blood samples were collected from the radial or femoral artery before heparin administration during elective diagnostic coronary cineangiography (blood samples were not collected after coronary cineangiography). The time points of blood collections are presented in **Fig. 1**. All samples were centrifuged at 4°C at 3000×g for 10 min, within 30 min after collection.

### Serum lipid measurements

Commercial enzymatic assay kits (Kyowa Inc., Tokyo, Japan) were utilized, and analysis was conducted using an automated analyzer, where the serum levels of total cholesterol (TC), high-density lipoprotein cholesterol (HDL-C) (a homogeneous method), and TG were determined. The levels of low-density lipoprotein cholesterol (LDL-C) were calculated using the Friedewald equation.^19)^ In the subset of the first 30 patients out of the 63 STEMI patients, the HTGL and LPL levels were measured in pre- and post-heparin serum using the HTGL-ELISA and monoclonal antibodies (MoAb mouse 9A1, MoAb rat 141A1), respectively.^20)^

### 3% PAGE

In this study, 3% PAGE was performed using a commercial diagnostic kit (Lipophor AS, Aska-labo, Kawasaki, Japan). A detailed description of this method was identified according to a previous report.^21)^ The lipoprotein profiles in the STEMI group were compared with those in the control groups. Pre- and post-heparin serum lipid profiles, HTGL and LPL in STEMI patients were compared. Delta % lipoproteins were calculated as follows: Delta % lipoprotein=(%lipoprotein in post-heparin serum)−(%lipoprotein in pre-heparin serum).

### Statistics

SPSS statistics version 15.0 (SPSS Inc., Chicago, IL, USA) was employed for data analyses. Continuous variables are expressed as mean±standard deviation in normally distributed variables. If the variables are not normally distributed, they are presented as median and interquartile range: median (25th and 75th percentiles). Student’s t-test or Wilcoxon’s signed-rank test was employed for group comparison of continuous variables with normal or non-normal population distributions, respectively. Categorized values were compared using the chi-squared test. P-value <0.05 was considered significant.

### Study approval

This research was conducted in accordance with the Declaration of Helsinki and was approved by the Institutional Review Board (approved number by Saitama International Medical Center IRB: 12-033). Informed consent was obtained from all patients.

## Results

The baseline characteristics and lipoprotein profiles in the pre-heparin serum in STEMI and control groups are presented in **Table 1**. The fasting time before blood sample collection in the STEMI group was similar to that in the control group. The very low density lipoprotein (VLDL) fraction in the STEMI group was not different from that in the control group. The fractions of intermediate-density lipoprotein (IDL) and high-density lipoprotein (HDL) in the STEMI group were significantly lower than those in the control group. Moreover, the fractions of low-density lipoprotein (LDL) and small-dense low-density lipoprotein (s-LDL) in the STEMI group were significantly higher than those in the control group.

**Table 1:** Table 1 Patient characteristics and lipoprotein profiles in the pre-heparin serum in the STEMI and control groups

	STEMI n=63	Control n=63	P-value
Age (year)	64±11	67±11	0.11
Sex male (%)	54 (86)	54 (86)	0.60
Height (cm)	163±8	165±8	0.23
Weight (kg)	64±13	64±11	0.89
BMI	24±4	23±3	0.39
History of			
Hypertension (%)	29 (46)	33 (52)	0.30
Diabetes mellitus (%)	21 (33)	10 (16)	0.04
Dyslipidemia (%)	21 (33)	9 (14)	0.01
Smoking (%)	47 (75)	46 (73)	0.50
Family history (%)	13 (21)	18 (29)	0.20
Systolic BP (mmHg)	145±37	129±16	<0.01
Diastolic BP (mmHg)	86±23	71±12	<0.01
Heart rate (/min)	77±19	72±12	0.09
Fasting time (hours)	7.3±3.7	8.1±3.0	0.22
TC (mg/dL)	207±44	191±36	0.06
TG (mg/dL)	103 (53,166)	119 (88, 192)	0.03
HDL-C (mg/dL)	49±17	55±15	<0.01
LDL-C (mg/dL)	132±38	106±33	<0.01
VLDL (%)	11 (6, 19)	11 (7, 16)	0.75
IDL (%)	0 (0, 7)	8 (6, 9)	<0.01
LDL (%)	60 (51, 67)	51 (46, 55)	<0.01
s-LDL (%)	2 (0, 4)	1 (0,1)	<0.01
HDL (%)	20 (16, 25)	27 (23, 33)	<0.01
HbA1c (%)	6.0±1.7	5.7±1.0	0.35
CRP (mg/dL)	0.10 (0.03, 0.26)	0.08 (0.04, 0.18)	0.11
Cre (mg/dL)	0.9±0.3	0.9±0.2	0.85

BMI: body mass index; BP: blood pressure; STEMI: ST-segment elevation myocardial infarction. Dyslipidemia refers to a patient who had pointed out dyslipidemia before hospital admission but had not received lipid-modifying medication. Cre: creatinine; CRP: C-reactive protein; HbA1c: hemoglobin A1c; HDL: high-density lipoprotein; HDL-C: high-density lipoprotein cholesterol; IDL: intermediate-density lipoprotein, LDL-C: low-density lipoprotein cholesterol calculated using the Friedewald equation; s-LDL: small, dense low-density lipoprotein; TC: total cholesterol; TG: triglyceride; VLDL: very-low-density lipoprotein

The serum lipid levels and lipase levels in the pre-and post-heparin serum in STEMI group are presented in **Table 2**. As can be seen from the table, HTGL and LPL significantly increased in post-heparin serum.

**Table 2:** Table 2 Lipid and lipase profiles in the pre- and post-heparin serum in STEMI group

	Pre-heparin	Post-heparin	P-value
TC (mg/dL)	207±44	185±38	<0.01
TG (mg/dL)	103 (53,166)	26 (17,37)	<0.01
HDL-C (mg/dL)	49±17	47±14	0.10
LDL-C (mg/dL)	132±38	128±40	0.04
LPL (ng/mL)	78 (45, 110)	264 (130, 521)	<0.01
HTGL (ng/ml)	2.2 (0.5, 7.3)	46.7 (24.2, 72.8)	<0.01

HDL: high-density lipoprotein; HDL-C: high-density lipoprotein cholesterol; HTGL: hepatic lipase; IDL: intermediate-density lipoprotein; LPL: lipoprotein lipase; LDL-C: low-density lipoprotein cholesterol calculated using the Friedewald equation; STEMI: ST-segment elevation myocardial infarction; TC: total cholesterol, TG: triglyceride

The cumulative relative frequency plots of s-LDL in the STEMI and control groups are presented in **Fig. 1a**, and those of IDL are presented in **Fig. 1b**. About 44% (28/63) of the STEMI group and 48% (30/63) of the control group were negative for s-LDL in pre-heparin serum. Moreover, 55% (35/64) of the STEMI group in pre-heparin serum is negative for IDL.

**Figure 2:**
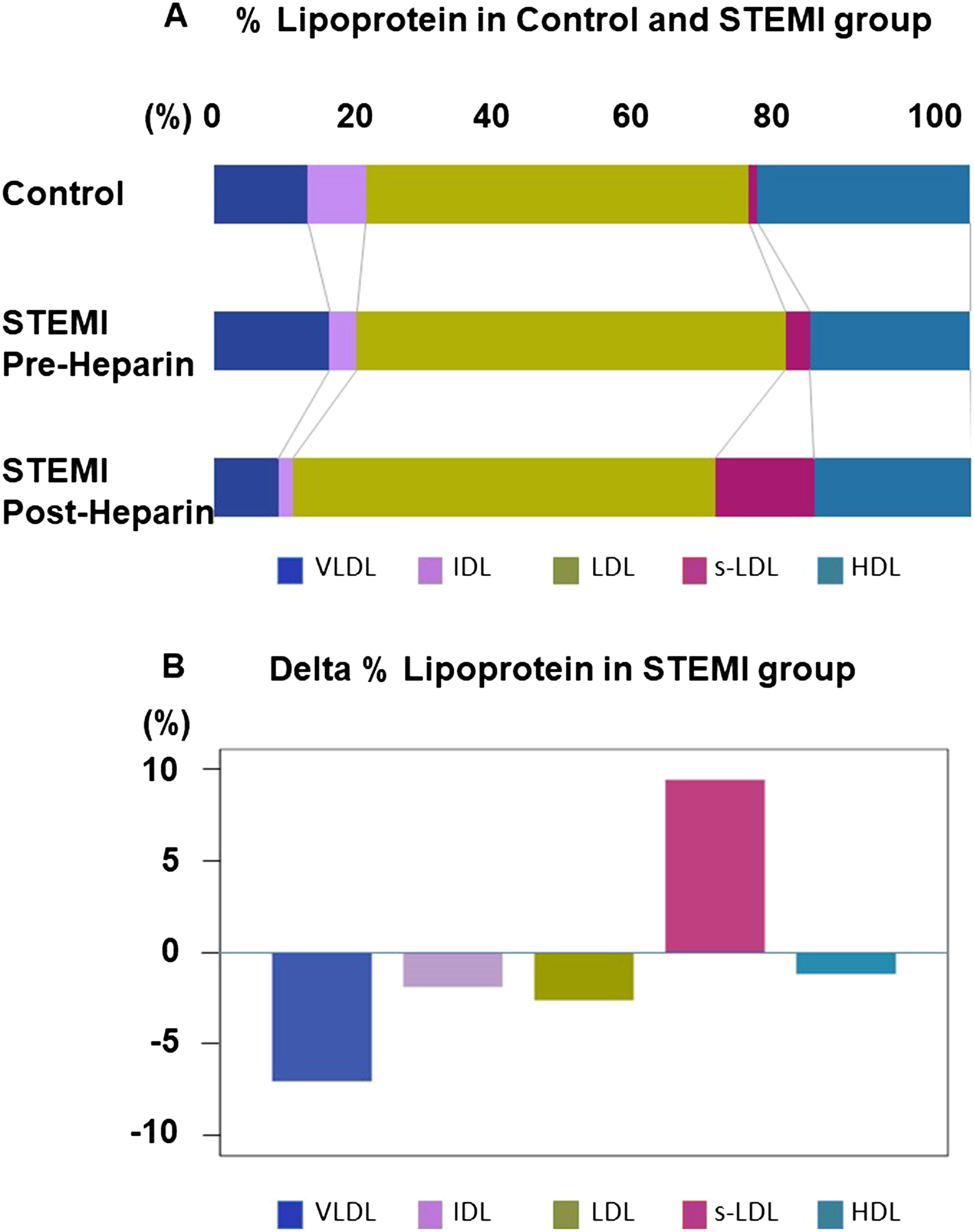
Fig. 2 (**A**) presents the distribution of lipoprotein profiles by 3% PAGE in the STEMI and control groups. (**B**) presents the delta % lipoproteins in the STEMI group. Only the fraction of s-LDL increased in post-heparin serum, whereas the other parameters decreased. Delta % lipoprotein=(% lipoprotein in post-heparin serum)−(% lipoprotein in pre-heparin serum).

HDL: high-density lipoprotein; IDL: intermediate-density lipoprotein; LDL: low-density lipoprotein; PAGE: polyacrylamide gel electrophoresis; s-LDL: small, dense low-density lipoprotein; STEMI: ST-segment elevation myocardial infarction; VLDL: very-low-density lipoprotein

The distribution of lipoprotein profiles in the control group and in the pre- and post-heparin serum in STEMI group are presented in **Fig. 2A**. The Delta % lipoproteins in STEMI group were ΔVLDL: −6 (−13, 0), ΔIDL: 0 (−6, 0), ΔLDL: −1 (−8, 0), Δs-LDL: 3 (0, 21), ΔHDL: 0 (−3, 2) (**Fig. 2B**). Note that the only increase observed in the post-heparin serum was that of s-LDL.

**Figure 3:**
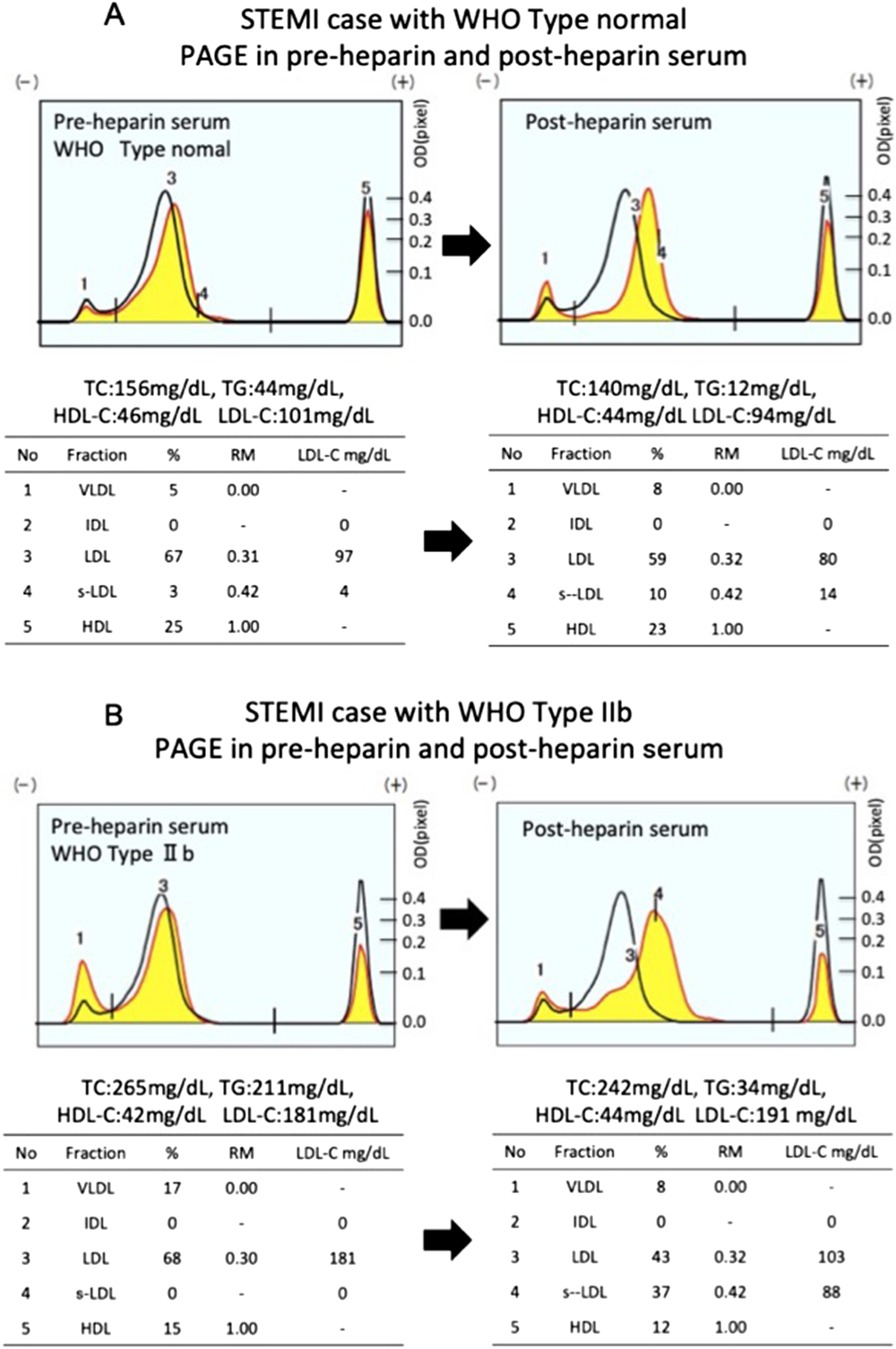
Fig. 3 (**A**) Representative densitometric patterns of PAGE in the pre- and post-heparin serum in two STEMI patients (normal lipid pattern and type IIb by the WHO classification). demonstrates the normal densitometric pattern of PAGE in pre- and post-heparin serum. Note that the peak of the highest yellow wave shifts to the right, indicating an increase in the s-LDL levels after heparin administration in vivo. (**B**) presents the type IIb densitometric pattern of PAGE in pre- and post-heparin serum. The peak of the highest yellow wave significantly shifts to the right, indicating an increased s-LDL level and a decreased VLDL level after heparin administration in vivo.

Representative densitometric patterns of PAGE in pre- and post-heparin serum in two patients with STEMI (normal lipid pattern: **Fig. 3A** and type IIb by the WHO classification: **Fig. 3B**) are presented in **Fig. 3**.

Black line: normal lipid control; yellow painting: subject; HDL-C: high-density lipoprotein cholesterol; IDL: intermediate-density lipoprotein; LDL-C: low-density lipoprotein cholesterol; OD: optical density; PAGE: polyacrylamide gel electrophoresis; s-LDL: small, dense low-density lipoprotein; STEMI: ST-segment elevation myocardial infarction; TC: total cholesterol; TG: triglyceride; VLDL: very-low-density lipoprotein

## Discussion

The principal findings of this study are as follows: 1) in the pre-heparin serum, the fraction of IDL in the STEMI group was significantly lower than that in the control group; 2) 44% of the STEMI group and 48% of the control group were negative for s-LDL in pre-heparin serum; and 3) in post-heparin serum, the fractions of VLDL and IDL significantly decreased, and that of s-LDL with the HTGL and LPL serum levels significantly increased, in concurrence with the large decline in the TG levels.

It has been reported that VLDL enhances platelet functions,^22)^ and an increased concentration of serum VLDL could account for the enhanced platelet activity observed in patients with hyperlipidemia. However, in the present study, the TC level at the onset of CAT was not statistically different from that in the control group, and the TG level was rather significantly lower than that in the control group. Thus, at the time of blood sample collection performed within 12 h from the symptom onset in STEMI group, VLDL may not be mainly associated with platelet activity leading to thrombus formation.

While previous studies^12)^ have confirmed the general consensus supporting that the significant decline in lipid levels is due to multiple reasons, including changes associated with acute phase reactions and initial metabolic and hormonal responses,^13)^ these explanations are not necessarily solid. Avogaro et al. revealed that serum TG levels are acutely reduced immediately after ACS, although the underlying mechanism of this decrease remains unclear.^14)^ The above results partially correspond with our present results, which indicate that the fraction of IDL and serum level of TG in the STEMI group are significantly lower than those in the control group. Previous studies have reported that lipoproteins activate platelets^23,24)^; circulating platelets may, in turn, have the ability to modify lipoproteins.^3)^ Considering the low profiles of lipoproteins, i.e., about half of the CAT patients are negative for s-LDL and IDL under conditions where platelets are activated, a hypothesis in terms of the causes of the low profiles of the lipoproteins in ACS has been proposed: circulating IDL and s-LDL may be rapidly consumed by substances secreted by activated platelets that facilitate macrophage take-up of lipoproteins at the beginning of CAT.^23–26)^ Considering that this hypothesis is still weak, we understand and concur that a more detailed analysis of lipoprotein profiles is important to evaluate the pathophysiology of CAT. Further studies are expected to prove this hypothesis. An additional study is required in this area.

### Lipoproteins in post-heparin serum

In the present study, the proportion of s-LDL significantly increased in post-heparin serum after primary PCI for STEMI. After heparin injection, the TG levels probably artificially decreased, and the fraction of s-LDL, levels of HTGL, and LPL increased. Of note, past reports^11–15)^ that quoted the lipid profiles in ACS also mentioned the possibility of an unexpected heparin effect on lipid profiles.

With regard to the findings of the present study that the fraction of s-LDL increased in the post-heparin serum in STEMI group, there is concern this rapid amplified s-LDL effect on thrombogenicity, that is, whether s-LDL plays a harmful role in, for example, thrombus formation in another pathway, owing to the reports that s-LDL particles probably enhance thromboxane synthesis and promote platelet aggregation.^27)^

### Study Limitations

First, this study presents a single-center experience with a small sample size. However, the blood samples were obtained before the administration of heparin in the emergency room, which is a very rare and valuable event that evaluates the raw lipid profile at the new onset of CAT in STEMI. Second, the questions that are of fundamental importance are to what degree the lipid levels are abnormal before CAT and to what extent the lipid pattern is changed by the thrombotic event. However, the lipid profile before the onset of CAT is not available; therefore, it is unclear whether the present lipid profile at the onset of CAT is only temporary. Furthermore, statin administration was initiated after PCI; consequently, the lipid profile after the STEMI therapy stabilization was modified by statins. Thus, the whole picture of the lipid profiles in the acute phase of STEMI was not observed in this study.

## Conclusion

Our results indicate that the lipoprotein profiles in patients with CAT are different from those in patients without CAT. Heparin widely modifies the lipoprotein profiles in patients with CAT leading to STEMI, indicating that the collection of blood samples before heparin administration is needed to better assess the lipoprotein profiles in CAT.
